# Added Value of Arterial Spin Labeling in Detecting Posterior Reversible Encephalopathy Syndrome as a Stroke Mimic on Baseline Neuroimaging: A Single Center Experience

**DOI:** 10.3389/fneur.2022.831218

**Published:** 2022-03-03

**Authors:** Joseph D. Weinstein, Omar Hamam, Victor C. Urrutia, Hanzhang Lu, Licia P. Luna, Aylin Tekes-Brady, Mona Bahouth, Vivek Yedavalli

**Affiliations:** ^1^Department of Radiology and Radiological Sciences, Division of Neuroradiology, Johns Hopkins School of Medicine, Baltimore, MD, United States; ^2^Department of Neurology, Comprehensive Stroke Center, Johns Hopkins School of Medicine, Baltimore, MD, United States; ^3^Neurofunction MRI Section, MR Research Division, Department of Radiology and Radiological Sciences, Johns Hopkins School of Medicine, Baltimore, MD, United States; ^4^Division of Pediatric Radiology and Pediatric Neuroradiology, Department of Radiology and Radiological Sciences, Johns Hopkins School of Medicine, Baltimore, MD, United States; ^5^Department of Neurology, Johns Hopkins School of Medicine, Baltimore, MD, United States; ^6^Division of Neuroradiology, Stroke Imaging, Department of Radiology and Radiological Sciences, Johns Hopkins School of Medicine, Baltimore, MD, United States

**Keywords:** arterial spin labeling, MR perfusion, stroke imaging, perfusion imaging, stroke mimic, PRES

## Abstract

Differentiating stroke from stroke mimics is a diagnostic challenge in every day practice. Posterior Reversible Encephalopathy Syndrome (PRES) is an important stroke mimic with nonspecific symptomatology, making prompt and accurate diagnosis challenging. Baseline neuroimaging plays a pivotal role in detection and differentiation of stroke from many common mimics and is thus critical in guiding appropriate management. In particular, MR perfusion (MRP) imaging modalities provide added value through detection and quantification of multiple physiological parameters. Arterial Spin Labeling (ASL) is a non-contrast, noninvasive MRP technique increasingly used in clinical practice; however, there is limited description of ASL in PRES in the existing literature. In this single center retrospective pilot study, we investigate the added value of ASL in detecting PRES in the largest series to date. We hope this study can serve as the basis for larger scale investigations exploring the utility of ASL in detecting stroke mimics such as PRES for accurate and efficient management of such patients.

## Introduction

Posterior reversible encephalopathy syndrome (PRES) is a common stroke mimic thought to be related to cerebrovascular dysregulation, resulting in blood-brain barrier disruption and vasogenic edema (1). This dysregulation can result in structural and perfusion abnormalities on neuroimaging that can assist in differentiating PRES–an otherwise often-challenging diagnosis–from ischemic stroke and other stroke mimics. This differentiation is critical in determining appropriate patient care.

MR perfusion techniques have significant utility in characterizing ischemic stroke and stroke mimics. Arterial Spin Labeling (ASL) is one such technique that has seen recent increased utilization and application in everyday practice (2). ASL has multiple advantages compared to the more commonly used dynamic susceptibility contrast MR perfusion (DSC-MRP) or CT perfusion (CTP) techniques in that it offers a repeatable, non-contrast, noninvasive imaging alternative without ionizing radiation. To date, few studies have explored or demonstrated the clinical utility of ASL in PRES.

The goal of our study was to characterize the patterns and frequency of abnormal ASL signal in clinically suspected or confirmed cases of PRES in order to identify the potential utility of ASL perfusion imaging in supporting the gold-standard clinical diagnosis.

## Methods

In this retrospective single-center experience, we reviewed 490 consecutive MRI brain examinations performed between July 2016 to March 2021 that all included standard structural imaging of the brain, time-of-flight MR angiography of the head (TOF MRA), and ASL perfusion. We subsequently excluded cases without a clinically suspected or confirmed diagnosis of PRES during the same hospital encounter based on chart review with particular attention to neurology documentation. We also excluded one case of suspected PRES because of significant motion degradation on ASL and multiple additional MR pulse sequences. In total, we identified six patients who qualified per our inclusion and exclusion criteria. Of note, gadolinium-enhanced dynamic susceptibility contrast (DSC) MR perfusion was not ordered as a part of the performed exams in any of the six patients who qualified for this study.

### Data Collection

Comprehensive data points on each patient were collected, including age, race/ethnicity, presence of seizure activity at or immediately prior to presentation, clinically suspected triggering etiology, systolic and diastolic blood pressure at initial presentation, and presence of corresponding MR signal abnormalities on ASL, FLAIR, and DWI sequences. Images were independently reviewed by a neuroradiology fellow (JW) and a fellowship-trained attending neuroradiologist with 3 years of experience (VY). Any conflicting interpretations were resolved by consensus review.

### Clinical and Radiologic Variables

The diagnosis of PRES was established clinically by the treating neurologist through evaluation of electronic health records. All patients underwent structural MR imaging of the head supplemented by MRA of the head and ASL perfusion imaging prior to the clinically confirmed diagnosis. ASL parameters at our institution were performed with 2D PASL (*n* = 5) or 3D PCASL (*n* = 1) labeling schemes on 3T Siemens scanners (2D PASL parameters: TR 2500, TE 12, Flip Angle 90, FOV 145.1 x 76.8 cm, SW 8; 3D PCASL parameters: TR 4550, TE 16, Flip Angle 90, FOV 43.5 x 23 cm, SW 5).

## Results

**Figure 1:**
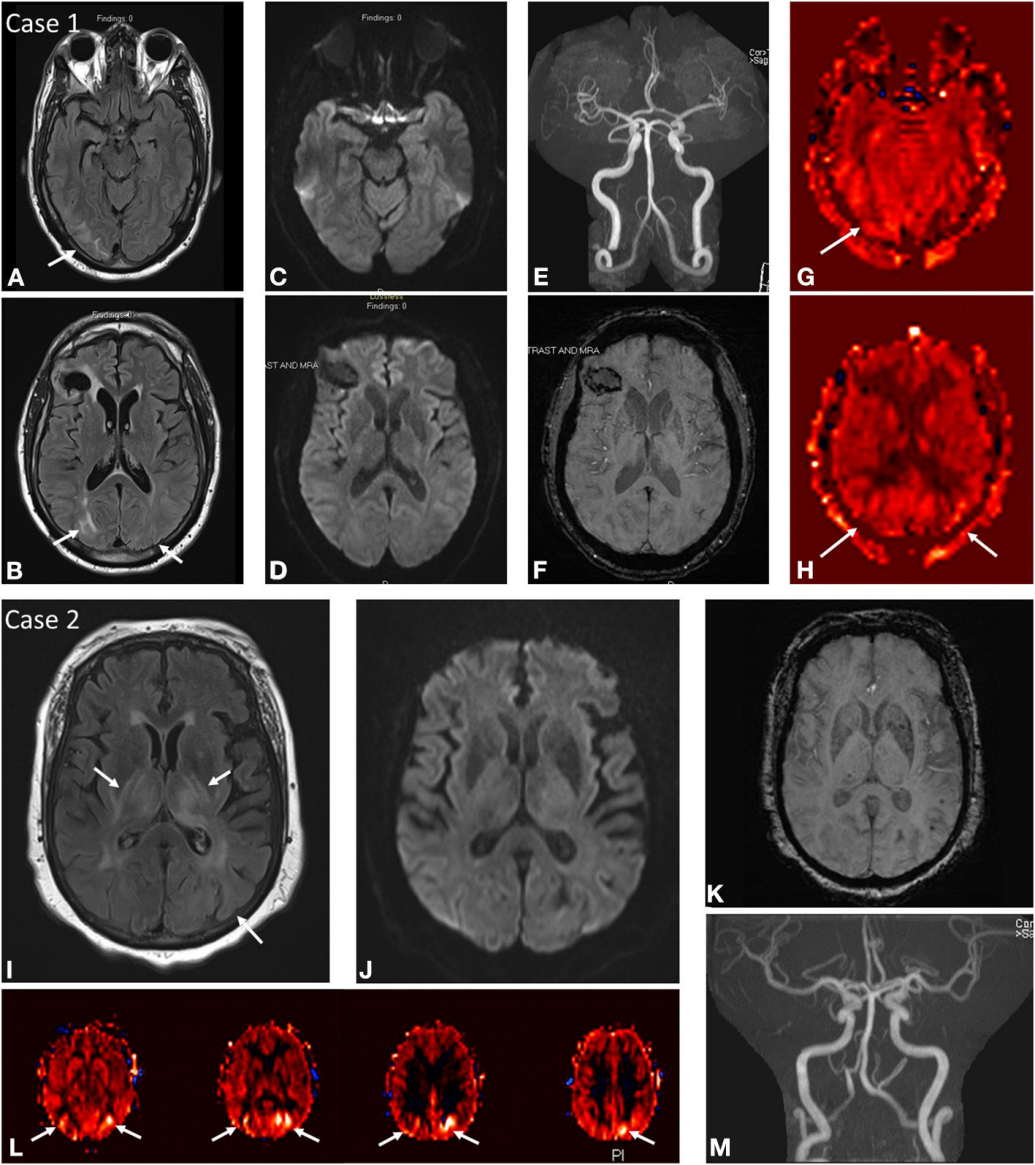
Case 1: A 45-year-old male brought in by ambulance after being found down with seizure activity in the setting of positive urine toxicology screen for cocaine and methamphetamines as well as severe hypertension of 235/101. Past medical history notable HIV on HAART, chronic Hepatitis C, ESRD on HD, and remote right frontal hemorrhage. MRI brain at initial presentation demonstrates abnormal FLAIR hyperintensity **(A,B)** in the subcortical white matter of the right greater than left occipital lobes with corresponding mixed ASL signal hyperintensity and hypointensity in these regions **(G,H)**. No DWI **(C,D)** or SWI **(F)** signal abnormality aside from remote right frontal hemorrhage. No focal arterial high-grade stenosis or occlusion on MR angiography **(E)**. Case 2: A 68-year-old female presenting with seizure activity in setting of past medical history notable for CKD, DM, HTN, HLD, CHF, and COPD. MRI brain at initial presentation demonstrates abnormal FLAIR hyperintensity **(I)** in the bilateral thalami, bilateral posterior limbs of the internal capsules, bilateral external capsules, and left putamen with more subtle signal hyperintensity in the subcortical white matter of the left occipital lobe. Corresponding ASL signal hyperintensity **(L)** in the left greater than right occipital lobes. No associated DWI **(J)** or SWI **(K)** signal abnormality in these regions, although the patient was noted to have a punctate acute infarct in the right temporo-occipital region (not shown). No focal arterial high-grade stenosis or occlusion on MR angiography **(M)**.

**Figure 2:**
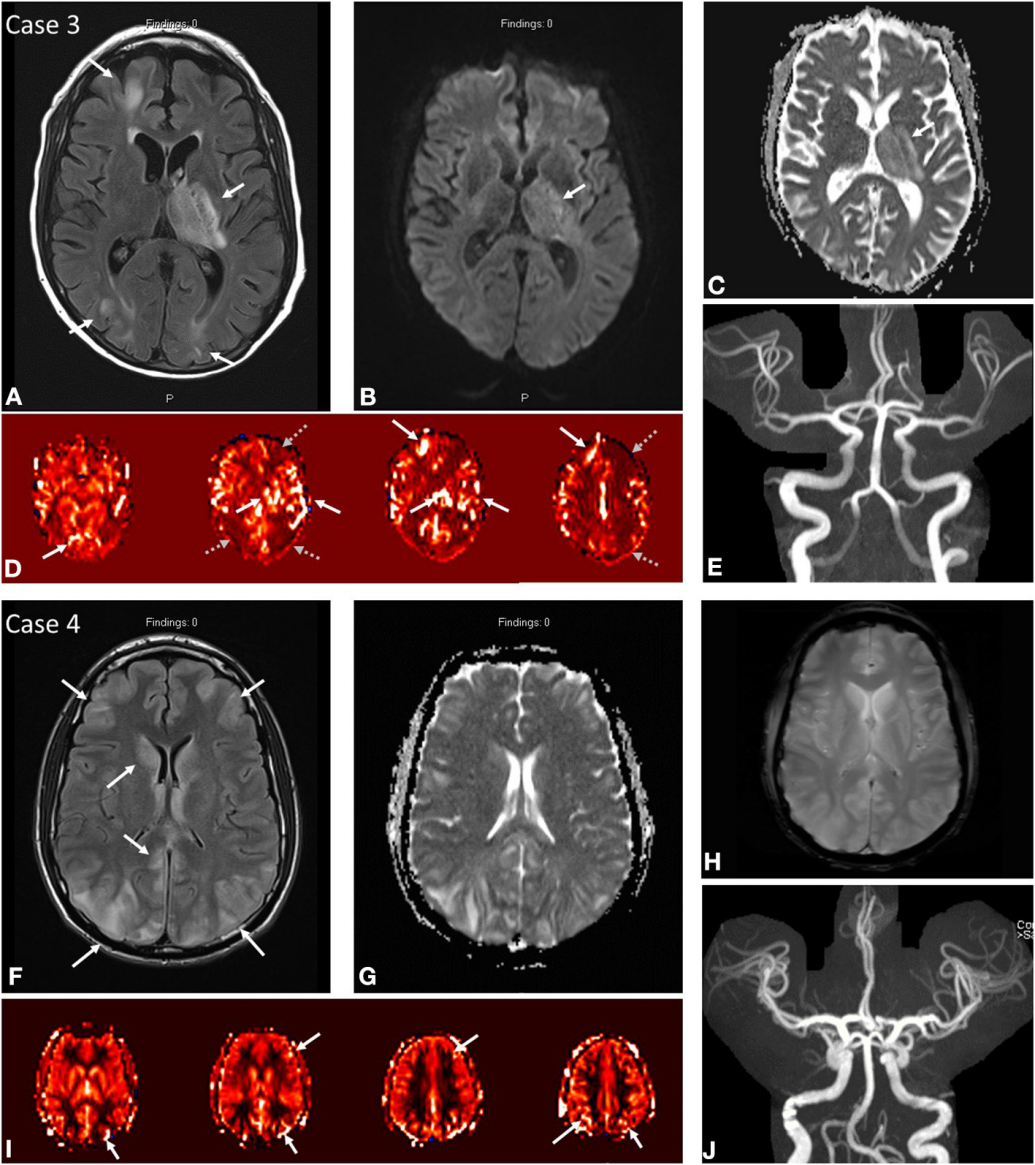
Case 3: A 53-year-old male presenting with seizure activity and past medical history notable for EtOH cirrhosis status post recent liver transplant on tacrolimus complicated by post-operative AKI on intermittent HD. MRI brain at initial presentation demonstrates abnormal FLAIR hyperintensity **(A)** in the left thalamus, posterior limb of the left internal capsule, and cortex/subcortical white matter of the left frontal and bilateral occipital lobes. DWI **(B)** and ADC **(C)** demonstrate predominantly T2 shine through in the left thalamus/internal capsule. ASL **(D)** demonstrates increased signal in the corresponding territories (solid white arrows) with notable severe border-zone hypoperfusion in the bilateral watershed territories (gray dashed arrows). No focal arterial high-grade stenosis or occlusion on MR angiography **(E)**. Case 4: A 21-year-old male presenting with seizure activity in setting of hypertension of 177/113 with past medical history notable for thrombocytopenia, oliguric renal failure on intermittent HD secondary to atypical hemolytic uremic syndrome, hemophagocytic lymphohistiocytosis on etoposide/steroids, and prior history of PRES. MRI brain demonstrates abnormal FLAIR hyperintensity **(F)** in the cortex and subcortical white matter of the bilateral frontal lobes and bilateral parieto-occipital regions with slight asymmetric signal hyperintensity in the right caudate nucleus. ASL **(I)** demonstrates increased signal in the corresponding bilateral frontal, parietal, and occipital regions. No DWI/ADC **(G)** or SWI **(H)** signal abnormality. No focal arterial high-grade stenosis or occlusion on MR angiography **(J)**.

**Figure 3:**
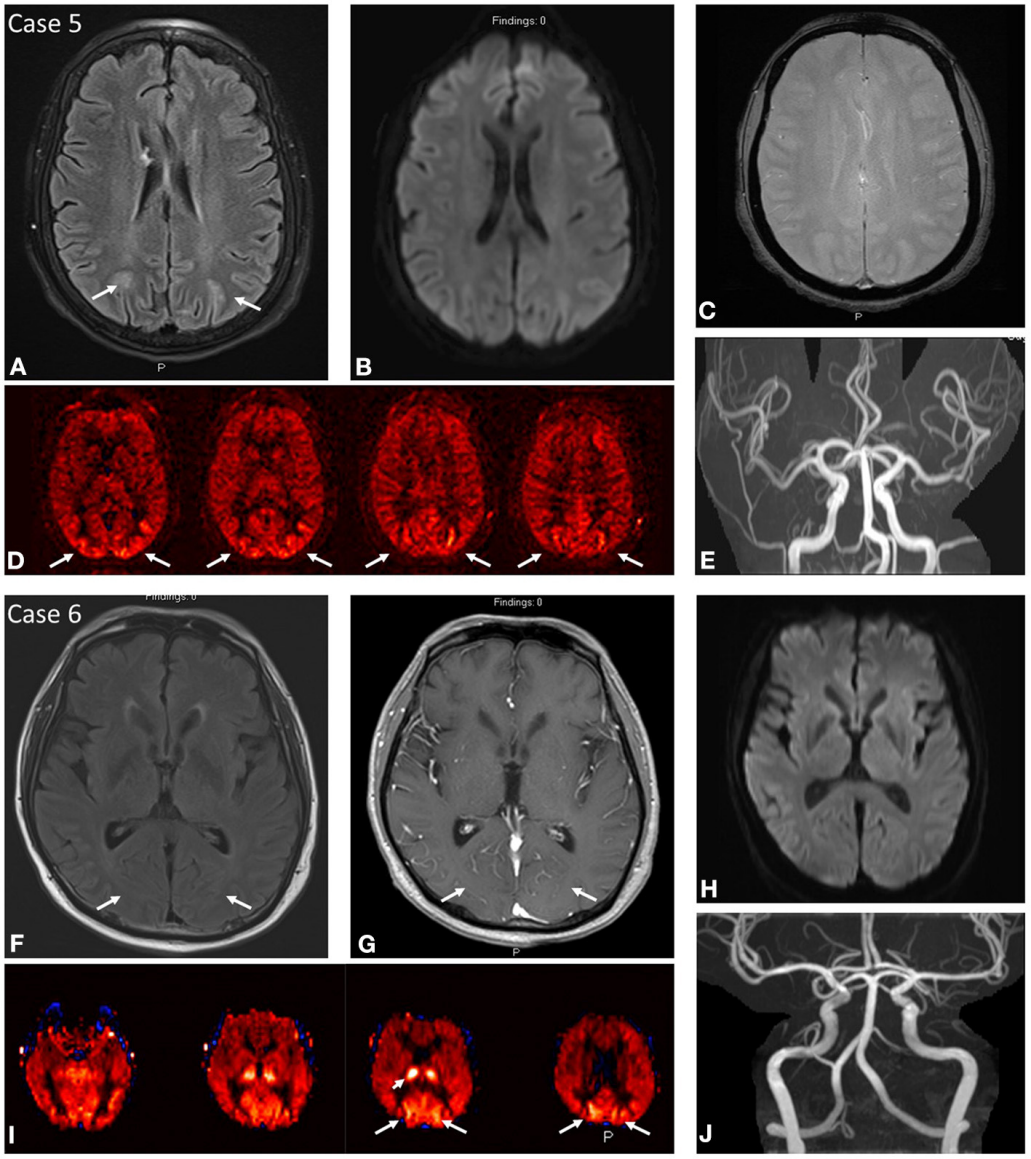
Case 5: A 64-year-old female presenting with uncontrolled hypertension of 205/93 and past medical history notable for sickle cell anemia. MRI brain at initial presentation demonstrates abnormal FLAIR hyperintensity **(A)** in the subcortical white matter of the bilateral parieto-occipital regions with corresponding regions of ASL signal hyperintensity **(D)**. No DWI **(B)** or SWI **(C)** signal abnormality. No focal arterial high-grade stenosis or occlusion on MR angiography **(E)**. Case 6: A 61-year-old male presenting with acute unresponsiveness in setting of prolonged hospital course due to COVID-19 pneumonia/ARDS with refractory hypoxemia and hypercarbia complicated by cardiac arrest, gastrointestinal bleeding, hypotension on pressors, and eventual bilateral lung transplant on tacrolimus. MRI brain demonstrates ASL signal hyperintensity **(I)** in the bilateral occipital lobes with asymmetrically increased signal in the right thalamus. Findings correspond to subtle FLAIR hyperintensity **(F)** with mild vascular congestion on post-contrast imaging **(G)**. No DWI **(H)** signal abnormality. No focal arterial high-grade stenosis or occlusion on MR angiography **(J)**.

Of the six cases, 66.7% (4/6) were male with an average age of 52 years (ranging from 21 to 68 years of age). Based on self-reported racial/ethnic data, 50% (3/6) identified as Black or African American, 16.7% (1/6) identified as White or Caucasian, 16.7% (1/6) identified as Asian, and 16.7% (1/6) declined to answer. At initial presentation, average systolic and diastolic blood pressures were 156 and 91, respectively, with two patients presenting with systolic blood pressures >200. In addition, 66.7% (4/6) demonstrated seizure activity at or immediately prior to presentation. Please see Table 1 for more detail. Of the imaging studies, 100% (6/6) demonstrated FLAIR signal abnormalities, 100% (6/6) demonstrated ASL signal abnormalities, 16.7% (1/6) demonstrated DWI signal abnormalities, and 0% (0/6) demonstrated focal arterial high-grade stenoses or occlusions on MR angiography. Of the ASL signal abnormalities identified, 83.3% (5/6) showed hyperperfusion (Figure 1, Case 2; Figures 2, 3) and 16.7% (1/6) demonstrated mixed ASL signal intensity (Figure 1, Case 1). Most patients exhibited multifactorial potential triggering etiologies: 66.7% (4/6) with AKI or CKD including three on intermittent HD, 50% (3/6) with hypertension including two with uncontrolled systolic blood pressure >200 on presentation, 33.3% (2/6) with history of organ transplantation on Tacrolimus, 16.7% (1/6) with COVID-19 infection, 16.7% (1/6) with sickle cell anemia, and 16.7% (1/6) with controlled HIV on HAART.

**Table 1:** Clinical characteristics of the six patients included in this study.

**Case**	**Age**	**Gender**	**Race/ethnicity**	**BP at presentation**		**Seizure activity**	**Signal abnormality**			**Magnet strength**	**ASL protocol**	**Clinical history**
				**Systolic**	**Diastolic**		**FLAIR**	**DWI**	**ASL**			
1	45	Male	White or Caucasian	235	141	Yes	Yes	No	↑/↓	3T	2D PASL	Uncontrolled hypertension, HIV on HAART, chronic Hepatitis C, CKD on HD)
2	68	Female	Black or African American	101	53	Yes	Yes	Yes	↑	3T	2D PASL	CKD, DM, HTN
3	53	Male	Declined to Answer	136	93	Yes	Yes	No	↑	3T	2D PASL	EtOH cirrhosis s/p recent liver transplant on Tacrolimus, post-operative AKI on intermittent HD
4	21	Male	Black or African American	177	113	Yes	Yes	No	↑	3T	2D PASL	Oliguric renal failure on intermittent HD secondary to atypical hemolytic uremic syndrome, and prior PRES
5	64	Female	Black or African American	205	93	No	Yes	No	↑	3T	3D pCASL	Uncontrolled hypertension, sickle cell anemia
6	61	Male	Asian	84	53	No	Yes	No	↑	3T	2D PASL	COVID-19 pneumonia status post bilateral lung transplant on Tacrolimus

## Discussion

Of our cohort of clinically suspected or confirmed cases of PRES with both structural and functional ASL perfusion imaging of the brain, we found that all patients (6/6) demonstrated both FLAIR and ASL signal abnormalities, suggesting that ASL offers additional perfusion imaging support to both structural imaging and the gold-standard clinical diagnosis. This is the largest series to date exploring the role of ASL in detecting PRES where most of the current literature is limited to case reports only. This concordance between ASL and FLAIR in our study lays the groundwork for future larger scale studies to further explore the robustness of this association.

Cerebral autoregulation is capable of maintaining constant blood flow to brain tissues despite fluctuations in systemic blood pressure and cardiac output. As previously described in the literature, PRES can demonstrate MR perfusion signal abnormality that can (1) be more extensive and (2) even precede signal abnormalities on FLAIR imaging (3). The concept of perfusion signal abnormality preceding FLAIR signal abnormality is rather intuitive given the consensus that alterations in cerebral autoregulation eventually result in cerebral vasogenic edema seen on FLAIR imaging. This notion is exemplified and reinforced by our findings in Case #6 in which the most conspicuous abnormality is identified on ASL perfusion imaging.

Hyperperfusion and hypoperfusion imaging patterns in PRES have been described in both CT perfusion and DSC MR perfusion, and multiple competing hypotheses exist to explain the variability seen in such advanced imaging techniques (4). The most often described explanation for hyperperfusion in PRES is felt to result from breakdown of cerebral autoregulation, often in the setting of severe hypertension, resulting in excessive blood flow, elevated capillary hydrostatic pressure, and subsequent vasogenic edema (3). This is exemplified in a case report by Hedna et al., which describes a patient with PRES presenting as a stroke mimic with uncontrolled hypertension and abnormal CT perfusion demonstrating increased cerebral blood volume (CBV), increased cerebral blood flow (CBF), and decreased time to peak (TTP) in the classic posterior cerebrovascular distribution (5). An alternative hypothesis for hypoperfusion in PRES suggests that compensatory vasoconstriction results in decreased blood flow/capillary perfusion pressure, eventual brain ischemia, and subsequent edema, possibly secondary to increased vascular permeability (3). Brubaker et al. examined MR DSC perfusion imaging in 8 patients with PRES and noted decreases in both CBV and CBF in the areas of characteristic FLAIR signal abnormality, supporting the hypoperfusion hypothesis; however, they also noted a lack of abnormal vascular permeability on K2 perfusion maps and very low rates of infarction/cytotoxic edema in most (7/8) patients (6). As a result, the authors hypothesized the possibility of both arterial and disproportionate venous vasoconstriction as a plausible explanation for elevated capillary hydrostatic pressure and vasogenic edema despite counterintuitively low perfusion parameters. In our case study, we identified 83.3% (5/6) with hyperperfusion on ASL imaging and 16.7% (1/6) with mixed ASL signal, primarily indicating support for the hyperperfusion theory although with notable lack of hypertensive history in 50% (3/6) of cases, raising the question of how the other variable risk factors may contribute to the underlying mechanism.

Prior literature has described sequential hypoperfusion and hyperperfusion phenomena in the same patient with PRES citing that the progression of imaging findings mechanistically suggested a loss of autoregulatory control, resulting in initial hypoperfusion, followed by arteriolar vasoconstriction to maintain perfusion pressures, followed by resultant rebound hyperperfusion and vasogenic edema (7). This variability in perfusion characteristics is much akin to perfusion imaging of hemodynamic changes observed in peri-ictal and post-ictal states of patients with recent seizure activity, which is a common presentation in patients with PRES (2). Despite the absence of clinically detectable seizure activity in 33% (2/6) of cases, both of those cases (#5 and #6) still demonstrated hyperperfusion pattern on ASL imaging, suggesting that the perfusion anomalies were more likely secondary to the underlying pathogenic mechanism rather than a consequence of the resultant seizure activity. While the mechanism is still incompletely understood, ASL remains a valuable tool in evaluating dynamic changes in cerebral perfusion secondary to its repeatable and quantifiable nature for evaluating trends within the same patient over time.

Additionally, while predominantly symmetric physiologic regional hyperperfusion has been described in the bilateral occipital lobes corresponding to visual cortex activation (8), our cases are notably distinct from this entity due to the asymmetrical signal abnormality and the strong correlation with signal abnormalities on structural imaging.

Our study has some limitations due to its retrospective nature and small case number due to the variable use of ASL perfusion imaging in routine practice outside of our designated MRI stroke protocol. Another potential difficulty with this study is the reference standard for the diagnosis of PRES. While PRES may be suspected based on patient history and risk factors, symptoms are often nonspecific and imaging is often heavily relied upon to suggest or “rule in” the diagnosis (3, 9). This reliance on imaging and the well-documented patterns on FLAIR imaging may tend to introduce a bias in favor of FLAIR positive cases. On the other hand, the lack of standardized ASL imaging protocols and unknown inter-reader agreement of ASL perfusion imaging in PRES both potentially limit the external validity of this technique in generalized practice. Nevertheless, in this single center experience, both FLAIR and ASL were positive in all six cases suggesting a correlation. ASL can potentially add value in cases where the FLAIR abnormality may be subtle (such as Case #6 in our series described above). Although our case series is the largest in the literature to date, larger-scale retrospective and prospective studies are necessary to further assess the strength of the correlation between ASL and structural imaging as well as the potential added value of ASL not only detection but also management of PRES.

## Data Availability Statement

The original contributions presented in the study are included in the article/supplementary material, further inquiries can be directed to the corresponding author.

## Author Contributions

JW: data collection, primary manuscript creation, and editing. LL, VU, HL, MB, and AT-B: editing. OH: editing and data collection. VY: idea generation, data collection, secondary manuscript creation, and editing. All authors contributed to the article and approved the submitted version.

## Conflict of Interest

The authors declare that the research was conducted in the absence of any commercial or financial relationships that could be construed as a potential conflict of interest.

## Publisher's Note

All claims expressed in this article are solely those of the authors and do not necessarily represent those of their affiliated organizations, or those of the publisher, the editors and the reviewers. Any product that may be evaluated in this article, or claim that may be made by its manufacturer, is not guaranteed or endorsed by the publisher.
